# Medical Jurisprudence in the Great Desert

**Published:** 1850-01

**Authors:** 


*Medical Jurisprudence in the Great Desert.*—An enterprising traveller, M. Eugene Daumas, ex-colonel of Spahis, who lately made a journey to the kingdom of Houssa, in the interior of Africa, found that surgery was there held in considerable estimation, and he furnishes some amusing instances of its importance in the decision of legal questions and family disputes.

In the city of Timimoun, it seems they give the following pithy injunction to the young bride on presenting her to her husband:—"Be silent as to his secrets. When he is joyous, do not let him see you sorrowful; and when he is sad, do not show yourself merry before him." But whether or not the young Arab ladies are in the habit of attending strictly to this precept, we are not told. We learn in the sequel, however, that if they are not particularly careful of their husband's secrets, they are well able, on occasion, to take care of their own.

Timimoun appears to be a city of some pretension in the Desert, as it contains five or six hundred houses, which being each built in its own garden, occupy a large space of ground. It is surrounded by a dry ditch, about a dozen feet deep, by seven or eight feet wide, and is enclosed by an embattled wall, on which are several small forts of two stories high, capable of containing thirty or forty combatants a-piece. Civilization here is about equal, the traveller considers, to what it was in Europe during the middle ages, or about a thousand years ago.

In this city we are told that surgery supplies the place of a penal code. If one individual wounds another, the surgeon is called in to estimate the damages, and these are assessed in proportion to the length and depth of the injury, which is ascertained by an instrument called the measure of blood. Questions of jurisprudence are also sometimes decided by an appeal to the faculty, of which the following anecdote is an instance:

A woman of the caste called Berbere (a wandering tribe), had married two husbands, withont letting either of them know that she had any other besides himself; for in the marriage contracts she had stipulated with one that he should never visit her, excepting between sunrise and sunset; and with the other, that he should never come till after nightfall, and should depart before daylight in the morning, by which arrangement they never met. Two different cadis had attested the agreements, and, thanks to the precautions taken, nothing disturbed for some time the harmony of this family compact.

"*Deux coqs vivaient en paix*," said La Fontaine. It was not a hen, however, in this case, which came to destroy their peace, but an infant—*eh voila la guerre allumee!* She wife of two husbands was in some perplexity, but she took heart, and revealed her expectations to both, when an explanation followed, and they were not a little confounded to find themselves officially in such a position towards each other.

"You are mad," said one; "this woman is my wife."

"She is mine, I tell you." said the other; "and it is you who should be pronounced mad!"

"You are neither of you mad," interposed the wife; "each of you is my husband—you have only .o observe the conditions of your agreements. Pray do not agitate me by your disputes, but await the event tranquilly."

However, a new altercation arose about the expected infant, and in order to have it decided to which of them it should belong, they at last agreed to refer the matter to the cadi.

After long deliberation—for the question was really perplexing—the worthy magistrate hit upon a solution of the difficulty; he decided that if the child were born during the day, it should belong to the husband of the day; if it were born after dark, it should belong to him of the night. This decision was very satisfactory, but it so happened that the disputed infant was born after sunset and before dark—that is, during the twilight hour, which belonged to neither husband, so that the decree of the cadi could not be put in execution. They then agreed to submit this new difficulty to the judgment of the marabout. The holy man listened to the pleadings, and ordered that the two husbands, the wife and the child, should all be brought before him, and at the same time he sent for the best surgeon in the city to attend with them.

When all were assembled, the marabout addressed the surgeon, and said, "Here are three egg shells of exactly equal size and weight; take two of them, and fill them with the blood of the husbands (one for each), then fill the third with blood from the infant." The doctor obeyed, and, after the operation was completed, the marabout ordered a pair of nicely balanced scales to be brought, in which were weighed separately the first two shells against the last. From this experiment it resulted that the blood of one of the husbands was found to be a trifle lighter than that of the child, and the other's was exactly of the same weight with it. On this being ascertained, the judge, turning to the latter, said, "In the name of God, I declare thee to be the father of this child. Take it away; it belongs to thee."

However ridiculous such a mode of arriving at a judgment may appear, it was at least decisive, and under such circumstances, it may be fairly doubted if a whole host of London magistrates could have settled the controversy in a more satisfactory manner.—*London Lancet.*

